# The Future of Biologics: Lessons from Hatch–Waxman

**DOI:** 10.1007/s43441-026-00961-9

**Published:** 2026-04-07

**Authors:** Brian J. Miller, Janet Woodcock

**Affiliations:** 1 Division of Hospital Medicine, Department of Medicine, Johns Hopkins University School of Medicine, Baltimore, MD USA; 2 Hoover Institution, Stanford, CA USA; 3 Brookeville, MD USA

## Abstract

The article review the challenges and lack of robust biologics market competition and drug pricing impacts, the history of the small molecule generics marketplace and the subsequent early evolution of the biosimilar marketplace. The article then explores the potential of an abbreviated biologics license application pathway to market—akin to the market entry pathway for generics—as a policy lever for improving biologics drug price competition, drug affordability and expanded access.

## Introduction

Prescription drug expenditures reaching $806 billion in 2024 [1] with biologic drugs comprising 49.6% of expenditures [2]. Biologic drugs also comprise a large share of specialty drug spending, a category accountable for much of the domestic drug cost growth. Specialty drug spending grew at an annual rate of 23.5% and now accounts for 71% of Medicare Part D spending [3]. The lack of biologic drug competition is problematic for specialty drug spending: 81% of biologic products or 216 products in 2022 had only 1 manufacturer whereas 18 had 4 or more manufacturers [4]. Biosimilar product development, a channel to promote price competition, is also stagnant with only 12 biosimilars in development for the 118 biologics expected to lose patent protection over the next decade [5].

Recent domestic drug affordability policy interventions focus on centralized administration pricing such as the Inflation Reduction Act and Most Favored Nation pricing. Remaining partially unaddressed and hampered by barriers to market entry in the form of product regulation and barriers to uptake in payment policy, the lack of vigorous biologic drug price competition highlights an opportunity to improve competition through an abbreviated biologic license application (ABLA) akin to the small molecule abbreviated new drug application (ANDA). This article reviews the history of the generics marketplace, the growth of the biosimilar marketplace, and the current opportunity to drive robust biologic drug price competition.

## The History of Small Molecule Generics Marketplaces

Prior to the creation of the ANDA pathway for market entry, generic product development lagged as manufacturers could either fully develop a copy to drive price competition or create a novel branded product to compete on non-price factors. Manufacturers generally chose competition between branded products on attributes such as efficacy, pharmacokinetic profile, or safety because the requirement to conduct clinical studies on a copy raised the costs of market entry.

As a result, price competition in small molecule generic markets lagged prior to the 1984 passage of the Drug Price Competition and Patent Term Restoration Act (Hatch–Waxman Act), with only 2 of 13 top drugs experiencing generic entry between 1976 and 1982 [6]. Hatch–Waxman represented a compromise that was revolutionary, unleashing price competition by tying market entry to a demonstration of pharmaceutical equivalence and bioequivalence. The scientific rationale was based on the ability to ensure that the generic product was the same chemical as the innovator product and achieved the same blood levels in the body, obviating the need to conduct new clinical and other studies.

The economics of generics markets were transformed. Contrasting the 1970s experience when clinical studies were required, from 1990–1993 an estimated 13 major drugs had patents expire with 11 having generic entry within 2 months of expiration [7]. Savings are significant, with massively lower development costs driving generic development and entry: FDA estimates savings from 2022 generic approvals totaled $18.9 billion in the subsequent year. Research demonstrates that generic price competition results in significant price decrements [8], impacts magnified through state substitution laws such that according to the FDA generic products account for 91% of all dispensed prescriptions. Researchers have also noted that in the context of robust price competition that regulatory and payment policy distortions coupled with contracting frictions can result in shortages [9, 10], highlighting the need for long term policy nuance when in promoting price competition in highly regulated markets.

## The History of the Biosimilars Marketplace

Although analytical techniques available in the late twentieth century were not sufficient to fully evaluate copies of proteins, investigators subsequently surmounted technical difficulties. In 2003 the European Union passed legislation enabling the European Medicines Agency to accept applications for “biosimilars”. Spurred both by Europe’s action and rising drug costs, in 2009 Congress followed suit, passing the Biologics Price Competition and Innovation Act (BPCIA). This legislation, like the European statute, enabled regulatory approval of copies of FDA-approved biotechnology products, but also contained an additional hurdle. While generic drugs benefit from automatic substitution at the pharmacy counter, under the BPCIA biosimilars in the U.S. (as initially was the case in Europe) would not be considered interchangeable unless they submitted additional data and studies beyond proof of biosimilarity, i.e., “switching studies.” Interchangeability for biologics is also more limited than the class-level substitution of generic product competition, allowing sole substitution of the interchangeable biosimilar—as opposed to all biosimilars—for a reference product. In 2015, the FDA approved the first biosimilar product, filgastrim-zarxio.

In addition to analytical comparisons of the reference (innovator) product to the candidate biosimilar the FDA advised product developers to undertake comparative clinical studies with pharmacokinetic, pharmacodynamic and clinical endpoints. Scientific market entry requirements raised clinical trial costs [11], with McKinsey reporting biosimilar development costing up to $300 million and 9 years.

Simultaneously, patients and physicians were reluctant to embrace biosimilars. Mirroring events subsequent to the passage of Hatch–Waxman, when some innovator firms spread concerns about the quality of generics [12] and many clinicians refused to prescribe them, after the introduction of biosimilars some physicians and their professional societies desired clinical studies showing equivalent performance. Interestingly, cognitive framing of “copycat” biologic products as inferior remains strong even in the face of evidence demonstrating reference product drift [13] and potential biosimilar superiority [14].

Recent economic impacts of biosimilar markets remain disappointing. In the intervening years since the passage of the BPCIA, the FDA approved 81 biosimilar drugs with 21 achieving interchangeability. After marketing approval, other market participants may modulate barriers to product adoption. Care delivery and health plan pharmacy and therapeutics committees can preference biosimilars for physician-administered drugs as part of formulary design while state substitution laws power uptake of the marketed interchangeable biosimilars for outpatient prescription drug markets [15]. Unfortunately, the small number of interchangeable products and high entry barriers has doomed the biosimilar market to repeating the early history of generic markets. Ensuring that older drugs become affordable by promoting price competition remains an untapped policy goal with studies demonstrating a 10–13% price reduction in originator for each biosimilar approved [16].

No population-level difference in the safety profile of US-approved biosimilars has been observed, with a systematic review finding no definitive harm from switching from the innovator to the biosimilar product [17]. Likewise, no approved biosimilar has been found to be less effective than the originator product.

Regulators have begun re-assessing requirements in light of these findings. In 2019, the FDA issued a draft guidance document discussing immunogenicity testing for biosimilar insulin products, suggesting that in most cases such testing would not be needed [18]. The FDA modernized the standards for interchangeability in 2024 suggesting that in many cases, analytical and comparative clinical studies showing comparability could obviate the need for switching studies [19]. Subsequently, in 2025 the FDA recognized through a new draft guidance document that a “Comparative Analytical Assessment (CAA) is generally more sensitive than a CES to detect differences between two products [20].”

Over a decade of regulatory and clinical experience has shown that an abbreviated development program can provide confidence that a proposed biosimilar product will have the same clinical performance as the innovator product. As with small molecule drugs, there will be exceptions. Topical drugs, products with unusually complex chemistry or formulations, and drug-device combination products have posed unique challenges to generic drug developers, preserving high prices for branded products. Similarly, there may be residual uncertainty about the performance of some biosimilars after an abbreviated program.

## Policy Actions to Advance Biologics Competition

The need for a more competitive biosimilars marketplace is clear. Regulators are taking an incremental approach to change and policymakers have begun to respond by first addressing product regulation barriers prior to adjusting payment policy. The proposed bipartisan Biosimilar Red Tape Elimination Act [21] would provide immediate interchangeability upon licensure for new biosimilars while the Expedited Access to Biosimilars Act [22] would require pharmacokinetic studies but eliminate blanket requirements for pharmacodynamic and immunogenicity unless warranted.

To meet the current imperative for robust competition and better patient access, a clear break from current practice should be taken. Congress should establish an abbreviated BLA pathway (ABLA) for biosimilars inclusive of class-level interchangeability with the reference product and instruct FDA to set up a review office independent of the new drugs program. An ABLA pathway should have mechanisms for determination of eligibility for the program, for adjudicating whether the development program has resulted in “residual uncertainty” about biosimilarity, and for deciding requirements to resolve such uncertainty.

Recognizing that some biosimilar candidates may encounter specific development challenges, an ABLA pathway should statutorily emphasize the minimum evidence required for bioequivalence while preserving regulatory flexibility. In order to improve patient and physician uptake and maximize the potential of an ABLA pathway, both physician groups and the FDA would need to undertake a public education campaign about biologic drugs and the growing role and potential of biosimilars.

While biosimilar developers would continue to encounter other obstacles such as patent challenges, payment policy barriers, and manufacturing infrastructure challenges, for the majority of biosimilar products an ABLA would be a more straightforward route to market and would attract more entrants, driving robust price competition, helping ensure that older drugs become and remain affordable for all Americans.

## Data Availability

No datasets were generated or analysed during the current study.
